# Respiratory infections in children up to two years of age on prophylaxis with palivizumab

**DOI:** 10.1590/0103-0582201432214813

**Published:** 2014-06

**Authors:** Ana Isabel M. P. Monteiro, Nancy Cristina J. Bellei, Alessandra Ramos Sousa, Amélia Miyashiro N. dos Santos, Lily Yin Weckx

**Affiliations:** 1 Escola Paulista de Medicina da Unifesp, São Paulo, SP, Brasil

**Keywords:** respiratory syncytial viruses, antibodies, monoclonal, respiratory tract infections/prevention & control, infant

## Abstract

**OBJECTIVE:**

To identify the viruses involved in acute respiratory tract infections and to analyze the rates of hospitalization and death in children on palivizumab prophylaxis.

**METHODS:**

Prospective cohort of 198 infants up to one year old who were born before 29 weeks of gestational age and infants under two years old with hemodynamically unstable cardiopathy or chronic pulmonary disease who received prophylactic palivizumab against severe respiratory syncytial virus infections in 2008. During the study period, in each episode of acute respiratory tract infection, nasopharyngeal aspirate was collected to identify respiratory syncytial virus, adenovirus, parainfluenza 1, 2 and 3, influenza A and B by direct immunofluorescence, rhinovirus and metapneumovirus by polymerase chain reaction preceded by reverse transcription. Data regarding hospitalization and deaths were monitored.

**RESULTS:**

Among the 198 studied infants, 117 (59.1%) presented acute respiratory tract infections, with a total of 175 episodes. Of the 76 nasopharyngeal aspirates collected during respiratory tract infections, 37 were positive, as follow: rhinovirus (75.7%), respiratory syncytial virus (18.9%), parainfluenza (8.1%), adenovirus 2 (2.7%), metapneumovirus (2.7%) and three samples presented multiple agents. Of the 198 children, 48 (24.4%) were hospitalized: 30 (15.2%) for non-infectious etiology and 18 (9.1%) for respiratory causes. Among these 18 children, one case of respiratory syncytial virus was identified. Two deaths were reported, but respiratory syncytial virus was not identified.

**CONCLUSIONS:**

During the prophylaxis period, low frequency of respiratory syncytial virus infections and low rates of hospitalization were observed, suggesting the benefit of palivizumab prophylaxis.

## Abstract (es)

**OBJETIVO:**

Identificar los virus implicados en los cuadros de infecciones agudas de trato respiratorio y analizar las tasas de internación y de óbito en niños sometidos a la profilaxis con palivizumabe.

**MÉTODOS:**

Cohorte prospectiva con 198 niños con menos de un año nacidas antes de 29 semanas de edad gestacional y niños con menos de dos años con cardiopatía hemodinámicamente inestable o enfermedad pulmonar crónica que recibieron palivizumabe para profilaxis contra infecciones graves por el virus sincitial respiratorio, en 2008. En el periodo de estudio, en cada episodio de infección aguda del trato respiratorio, se recogió aspirado de nasofaringe para identificar virus sincitial respiratorio, adenovirus, parainfluenza 1,2 y 3, influenza A y B por técnica del anticuerpo fluorescente directa, rinovirus y metapneumovirus por reacción en cadena de la polimerasa precedida por transcriptasa inversa. Se monitorean internaciones y óbitos en ese grupo.

**RESULTADOS:**

De los 198 niños seguidos, 117 (59,1%) presentaron infecciones agudas de trato respiratorio, totalizando 175 episodios. De 76 aspirados de nasofaringe recogidos en la vigencia de infecciones de trato respiratorio, 37 fueron positivos, encontrándose: rinovirus (75,7%), virus sincitial respiratorio (18,9%), parainfluenza (8,1%), adenovirus (2,7%), metapneumovirus (2,7%) y múltiples agentes en tres muestras. De los 198 niños, 48 (24,4%) fueron internados, siendo 30 (15,2%) por etiología no respiratoria y 18 (9,1%) por problemas respiratorios; entre los 18 casos, uno fue por virus sincitial respiratorio. Dos niños evolucionaron a óbito, no habiendo sido identificado el virus sincitial respiratorio.

**CONCLUSIONES:**

En la vigencia de profilaxis, se observó frecuencia baja de infecciones por el virus sincitial respiratorio y bajo índice de hospitalizaciones, sugiriendo beneficio de la profilaxis con palivizumabe.

## Introduction

Respiratory syncytial virus (RSV) is the major cause of acute lower respiratory tract infections in children younger than 2 years of age. It has a global distribution and a seasonal behavior. It is estimated that, worldwide, 33.8 million new episodes of acute lower respiratory tract infections by RSV occur annually in children under 5 years of age. Among these, about 3.4 million require hospitalization and 66 to 199 thousand children evolve to death, 99% occurring in developing countries^(^ 1 ^)^.

In general, primary RSV infection evolves as a common cold. However, approximately 25% of children under 2 years may present, in its first episode, infection of the lower airways, severe respiratory failure, need for ventilatory support, and, occasionally, death^(^ 2 ^)^.

Preterm children with chronic pulmonary disease, immunocompromised children, and children with heart diseases (particularly cyanotic) present greater risk of severe respiratory disease by RSV^(^ 3 ^-^ 6 ^)^. Some environmental factors are also associated with higher incidence of RSV infections, such as exposure to smoke, attendance at day care centers, or household contact with other children^(^ 4 ^,^ 5 ^)^.

In tropical and subtropical countries, the occurrence of RSV infections is greater in fall and winter, with significant regional variations^(^ 7 ^)^. Epidemiological studies on RSV in the Southeast region of Brazil showed that the infection by RSV starts in April, with peaks in May and June^(^ 2 ^,^ 8 ^)^. More recent studies conducted in the municipality of São Paulo showed that the RSV season occurs from March to July, and it may start earlier or last up until August^(^ 9 ^,^ 10 ^)^.

There is no specific treatment for RSV infection^(^ 7 ^,^ 11 ^)^, and prophylaxis with a specific anti-RSV monoclonal antibody (palivizumab) is widely recommended for high-risk cases^(^ 4 ^-^ 6 ^)^. Its use demonstrated a reduction in the rates of hospitalization by RSV infection (55%), hospitalization in ICUs (57%), length of hospital stay (42%), and the number of days with need of oxygen by 40%^(^ 4 ^,^ 5 ^,^ 12 ^,^ 13 ^)^.

Palivizumab is, however, a high cost medication. In Brazil, until 2007, many children received this medication after judicial demands, not always based in technical criteria^(^ 14 ^)^. The resolution SS n. 249, from July 13, 2007, from the Health Secretariat of the State of São Paulo^(^ 15 ^)^, made palivizumab freely available for SUS (Brazilian public Unified Health System) patients who were classified as "highly recommended" by the Brazilian Pediatrics Society^(^ 5 ^)^. Some centers were indicated by the State Secretariat as a place for the application of the palivizumab and the Reference Center for Special Immunobiologicals (*Centro de Referência para Imunobiológicos Especiais* - Crie) from Universidade Federal de São Paulo (Unifesp) was the first center designed for the municipality of São Paulo.

In this context, the objectives of the present study were to describe the etiology of acute viral respiratory infection in children on prophylaxis with palivizumab and to analyze the rates of hospitalizations and deaths in this group of children.

## Method

This was a prospective study with infants who received palivizumab at the Crie-Unifesp in 2008. The project was approved by the Research Ethics Committee of the institution under n. 0342-08, and the signature of the informed consent by parents/guardians was required.

The study included infants with indication of palivizumab according to the normative resolution SS n. 249, from July 13, 2007^(^ 15 ^)^, that is, children younger than 1 year of age, born with gestational age lower than 29 weeks, after hospital discharge, and children younger than 2 years, with hemodynamically unstable cardiopathy or chronic pulmonary disease, who needed treatment in the 6 months prior to the period of the RSV season.

Prophylaxis started in April 2008, including infants who received the first dose of palivizumab until the last month of June, in the dosage of 15mg/kg, via intramuscular (IM), in the vastus lateralis muscle of the thigh, with an interval of 30 days and maximum of five doses. The scheduling was performed grouping patients twice a week to share the flask of the immunobiological.

Data on the clinical records of the mother and the child and the reason for indication of the anti-RSV prophylaxis were collected on the day of the first dose of palivizumab by means of a questionnaire applied to the parents, besides the analysis of a medical form that indicated the administration of the monoclonal antibody.

Patients were followed with monthly visits during all the season (April to September), and the parents were instructed to communicate the researcher in case the child presented acute respiratory tract infection during this period (passive vigilance). Furthermore, each patient was contacted by phone once a week to search for respiratory intercurrences and hospitalizations (active vigilance) during the entire study period.

The acute respiratory tract infection was defined according to the World Health Organization^(^ 16 ^)^ as the presence or not of fever, associated to running nose or congestion, sore throat, cough, sibilance, tachypnea or respiratory difficulty.

In the presence of acute respiratory symptoms, a nasopharyngeal aspirate (NPA) was collected until the fourth day after the start of symptoms for the investigation of the following respiratory viruses: influenza A and B, parainfluenza 1, 2, and 3, adenovirus, RSV, rhinovirus, and metapneumovirus. For these patients, a second questionnaire was applied with clinical data on the current respiratory event.

The RSV hospitalization rate was calculated using the rate of hospitalization for respiratory distress, multiplied by the number of children testing positive for RSV, divided by the number of children hospitalized for respiratory infection who underwent collection^(^ 13 ^)^.

The collection of the nasopharyngeal aspirate was conducted with probe n. 6 or 8, inserted in the nasopharynx after washing with saline solution at 0.9%. The aspirate was placed in a sterile flask with 5mL of saline solution at 0.9%. The sample was kept under refrigeration at 2-8°C and brought to the Laboratory of Virology of the institution in 2 hours maximum. 

The samples were fractionated, and an aliquot was centrifuged to perform the direct immunofluorescence and the other, was frozen at -80°C, for molecular testing. We performed direct immunofluorescence for influenza A and B, parainfluenza 1, 2 and 3, adenovirus, and RSV, according to the Light Diagnostics^(r)^ Respiratory Screen Kit - Respiratory Panel 1 DFA (Chemicon International, Inc.). All samples underwent polymerase chain reaction preceded by reverse transcriptase for rhinovirus and human metapneumovirus^(^ 17 ^-^ 19 ^)^.

Numerical variables with normal distribution were compared by Student's *t* test and those with abnormal distribution, by the Mann-Whitney test. Categorical variables were compared by chi-square or Fisher's exact test. Statistical analysis were performed with the Statistical Package for the Social Sciences (SPSS), version 17, and significance was established at *p*<0.05. 

## Results

In 2008, 210 infants underwent prophylaxis with palivizumab at Crie-Unifesp. Among these, nine were excluded for not meeting the inclusion criteria, one for not having a telephone contact and two because they were transferred to another service before the end of the prophylaxis period. Thus, a total of 198 infants were included in the study.

The children assessed presented mean gestational age of 30.2±5.1 weeks (range of 23.0-41.2), birth weight of 1,482±900g (range of 450-4,165), median duration of mechanical ventilation of 12 days (range of 0-163; Q1 [1st quartile]-Q3 [3rd quartile]: 2-40 days) and remained at the neonatal unit for 76±60 days (range of 2-638; Q1-Q3: 48-100 days). The mean age range of infants at the moment of inclusion in the study was of 8.4±4.9 months, being 52% male.

Among included children, 142 (71.7%) were born in a private hospital and 56 (28.3%), in a public hospital. The median household budget was of R$ 2,500.00 (range of R$ 300.00-20,000.00; Q1-Q3: R$ 1,050.00-5,000.00) with the following social classification, according to the Brazilian Population Studies Association (ABEP - criterion Brazil 2008): class A1 - 5 (2.5%) families; A2 - 34 (17.2 %); B1 - 38 (19.2%); B2 - 57 (28.8%); C1 - 27 (13.6%); C2 - 29 (14.7%) and D - 8 (4.0%) families.

The median number of adults in the household was equal to 2 (range of 0-7), of adolescents, 0 (range of 0-2), and of children, 1 (range of 1-4). The number of households with smokers was of 57 (28.8%), with 22 (11.1%) smoking mothers.

Based of the criteria of indication for palivizumab, 29 (14.6%) children presented gestational age <29 weeks without bronchopulmonary dysplasia; 53 (26.8%) were hemodynamically unstable neonates with congenital heart disease, 116 (58.6%) presented bronchopulmonary dysplasia, and 91 (78.4%) preterm infants with dysplasia presented gestational age <29 weeks.

Each child received a median four doses of palivizumab (variation of 1-5; Q1-Q3: 3-4 doses), totaling 754 doses, using 749.1mL of the immunobiological. Among the 198 children followed, 45 (22.7%) received five doses of palivizumab, 75 (37.9%) four doses, 75 (37.9%) three doses, 1 (0.5%) received two doses and 2 (1.0%) received one dose. The interval between the doses was less than 35 days in 96.9% of cases from the first to the second dose, in 93.3% of cases from the second to the third dose, in 78.3% of cases from third to fourth dose, and in 60.0% of cases from fourth to fifth dose.

**Figure 1:**
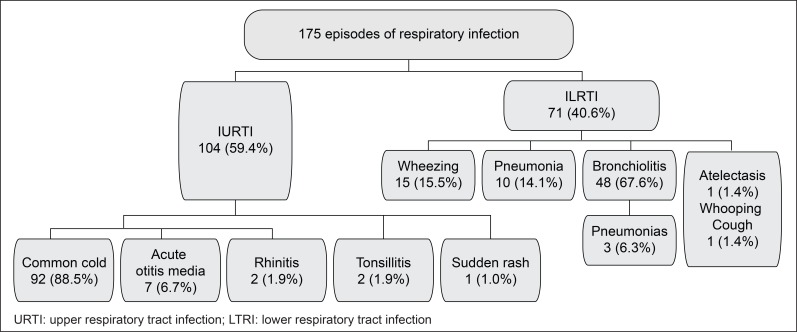
Incidence of upper respiratory tract infection and lower respiratory tract infection in infants who were on palivizumab prophylaxis, from April to September 2008

In relation to clinical events, 117 (59.1%) children had at least one episode of respiratory infection, totaling 175 episodes with a median of 1.0 (range of 0-4; Q1-Q3: 1-2 episodes/patients) and mean of 1.5 episodes/patient. Among respiratory infections, 104 (59.4%) were of the upper respiratory tract and 71 (40.6%), of the lower respiratory tract (Figure 1).

Among the children studied, 48 (24.2%) required hospitalization, being 30 (15.2%) due to non-respiratory etiology and 18 (9.1%) for respiratory problems. Of the 18 admissions for respiratory causes, nine (50%) were due to pneumonia and nine (50%) for bronchiolitis, and, of these, three progressed to pneumonia. The median length of hospitalization for these diseases was 9 days (range of 3-28; Q1-Q3: 4-12 days).

NPAs were collected in 13 (72.2%) of the 18 hospitalized cases, of which 11 were negative (six cases of bronchiolitis and five cases of pneumonia), in one case RSV was detected (bronchiolitis) and, in another, parainfluenza 2 and rhinovirus (bronchiolitis) were identified. The RSV hospitalization rate was of 0.7% (18/198 *versus* 1/13), according to calculation described above.

The comparison between children with and without bronchiolitis showed no statistical difference regarding sex, birth weight, gestational age, length of stay in the nursery, days of mechanical ventilation, attendance at daycare centers, and presence of smokers in the household. However, children with bronchiolitis had lower rates of breastfeeding (*p*=0.021) and more hospitalizations (*p*<0.001) (Table 1).

**Table 1:**
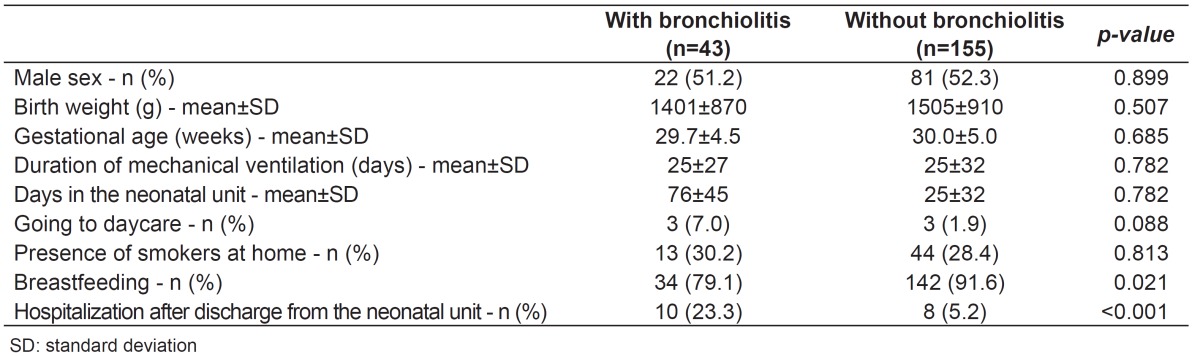
Demographic and clinical characteristics of the subjects, according to the presence of a diagnosis of bronchiolitis

At follow-up, there were two (1%) deaths, one in the postoperative period of cardiac surgery (hypoplastic left ventricle) and the other after hospitalization for pneumonia in a child with congenital heart disease who progressed to septic shock. In this patient, nasopharyngeal secretion was negative for the viruses surveyed.

**Figure 2:**
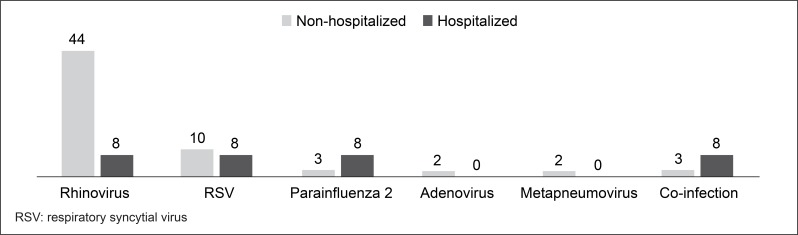
Distribution in percentage of viruses found in nasopharyngeal aspirates in hospitalized and non-hospitalized infants with signs of airway infection

Of the 175 episodes of respiratory infection, 76 (43.4%) NPAs were collected, being 37 (48.7%) negative; in two patients, the collected material was insufficient and, in 37 (48.7%), at least one virus was detected. The viruses identified are shown in Figure 2. Among the seven cases in which RSV was detected, two occurred in April, three in May, and two in July.

## Discussion

This is the first Brazilian study that assessed respiratory infections and involved viruses in children who received RSV prophylaxis with palivizumab.

Of the 198 children followed during the study period, about 60% had at least one episode of respiratory infection, with a prevalence of acute infections of the upper respiratory tract, being the common cold the most frequent one. Bronchiolitis was the most frequent lower respiratory tract infection. Regarding the NPA samples collected in the presence of respiratory infection, in half of the cases some sort of virus was detected, being the rhinovirus the most frequent, followed by the RSV. In the cases where rhinovirus was identified, about 1/3 presented acute lower respiratory tract infections with wheezing episode, confirming the literature data^(^ 20 ^)^ that show the rhinovirus as an important cause of wheezing in children under 2 years old. Of the seven children with RSV, six (85.7%) had bronchiolitis and one, a common cold. No case of influenza was detected. Children over 6 months and their parents received the influenza vaccine in the season evaluated.

Among children hospitalized for respiratory infection, we observed a low rate of hospitalization for rhinovirus, and only one case of bronchiolitis with rhinovirus coinfection and parainfluenza 2, unlike what was reported by Piotrowska et al, who found a hospitalization rate of 55% among cases where rhinovirus was detected^(^ 21 ^)^.

Studies on the incidence of RSV include children with a wide variability in age, region, community, or hospital population. This fact, coupled with the lack of Brazilian studies with cohorts of children at high risk for use of palivizumab, hinders the comparison with the data found in this investigation.

In this study, the rate of hospitalization for respiratory disease in children who received palivizumab was 9.1% and the rate of hospitalization for RSV was of 0.7%. These data show low rates of RSV hospitalization in the cohort who received palivizumab, compared with studies in children at high risk for severe RSV infection without prophylaxis. A classic multicenter study with preterm infants at high risk observed a rate of hospitalization for RSV of 10.6% in the group without prophylaxis, with a 55% reduction in these rates on the group with prophylaxis^(^ 12 ^)^. In another study with children under 2 years with congenital heart disease with hemodynamic consequences and not receiving prophylaxis, the rate of hospitalization for RSV was of 9.7%^(^ 3 ^)^. When analyzing studies of high-risk children who received palivizumab, our findings are comparable. Boivin et al^(^ 22 ^)^ found hospitalization rates of 12.9% for acute respiratory tract infections and 0.9% for RSV in a group of children on prophylaxis with palivizumab. Paes et al^(^ 23 ^)^, who analyzed children on palivizumab from 2005 to 2012 in Canada, observed a rate of hospitalization for acute respiratory tract infections of 6.6% and, for RSV, of 1.5%. 

In this study, the cases of infection by RSV occurred between April and July. Epidemiologic data show that, in São Paulo, when the prophylaxis was initiated in April, the RSV was already in circulation, what suggests that the prophylaxis should be initiated earlier. Also, studies with children hospitalized in the municipality of São Paulo showed increased frequency of RSV infections from March to July^(^ 9 ^,^ 10 ^)^. Among the 198 children followed, 98.5% received three or more doses of palivizumab, with an adequate interval between the doses until the third dose. However, there was a delay, on average, of 6.6 days (range of 1-15) in the interval between the fourth and fifth doses, due to delay in the supply of palivizumab by the Heath Department of the state of São Paulo in August. Children who were discharged form the neonatal unit after the beginning of the season received a lower number of doses, as well as those who were hospitalized during the period of prophylaxis, once palivizumab was available only for children who were not hospitalized. Thus, if prophylaxis with palivizumab were initiated at the correct season also for preterm infants hospitalized in the neonatal unit, as recommended by the Brazilian Society of Pediatrics^(^ 24 ^)^, children could have received a greater number of doses. 

The optimization strategy of the administration of palivizumab with grouped scheduling led to an economy of 28.2%, with the use of 749.1mL of palivizumab, in comparison to 1,044mL that would have been used in case of individual scheduling^(^ 25 ^)^. 

Despite having included many children, it was observed that most came from private services, probably due to increased access to information of these families and of the private services, showing the need to improve the dissemination of the program in all health services. This was the first year of RSV prophylaxis in the state of São Paulo and, according to data of the Immunization Division of the Health Department of the State of São Paulo, in 2010, the absolute number of patients enrolled in the program nearly doubled, with an equitable origin between public and private services^(^ 26 ^)^.

In May 2013, a new decree from the Health Ministry approved the protocol for the use of palivizumab in the entire national territory, respecting the seasonal differences of each region. Targeted children were preterm infants with less than 1 year old, born with gestational age lower or equal to 28 weeks, besides children with up to 2 years old with chronic pulmonary disease or congenital heart disease with proved hemodynamic repercussion. They also included hospitalized children, administering palivizumab, in these cases, in the hospital environment^(^ 27 ^)^.

The lack of a control group was a limitation of this study; however, it would be unethical to include a group of children of the same age range, with similar characteristics, without providing the monoclonal antibody. It was also not possible to collect NPA from all cases of respiratory infections, especially in cases of acute infections of the upper respiratory tract. There was a limitation in performing laboratory tests on weekends, hampering collection, especially in cases of short-duration respiratory infections or because of family travel.

Therefore, in seasons of high circulation of RSV, the palivizumab prophylaxis program in high-risk children showed low frequency of RSV infections, with a low rate of hospitalization and no deaths by this agent, what suggests the advantage of the palivizumab prophylaxis. In children treated with palivizumab, the main isolated virus in acute respiratory tract infections was the rhinovirus, with high rates of wheezing and low hospitalization rates, followed by RSV.
