# Supplementary material for: Respiratory infections in children up to two years of age on prophylaxis with palivizumab
Source: Rev Paul Pediatr. 2014 Jun;32(2):152–8. doi: 10.1590/0103-0582201432214813 (PMC4183017; doi:10.1590/0103-0582201432214813)
Supplement: Translation

# Translation: Infecções respiratórias em crianças menores de dois anos de idade submetidas a profilaxia com palivizumabe

**Authors:** Ana Isabel M. P. Monteiro, Nancy Cristina J. Bellei, Alessandra Ramos Sousa, Amélia Miyashiro N. dos Santos, Lily Yin Weckx

**Affiliations:** 1 Escola Paulista de Medicina da Unifesp, São Paulo, SP, Brasil

**Keywords:** vírus sinciciais respiratórios, anticorpos monoclonais, infecções respiratórias/prevenção & controle, lactente

## Abstract

**OBJETIVO:**

Identificar os vírus envolvidos nos quadros de infecções agudas de trato respiratório e analisar as taxas de internação e de óbito em crianças submetidas à profilaxia com palivizumabe.

**MÉTODOS:**

Coorte prospectiva com 198 crianças menores de um ano nascidas antes de 29 semanas de idade gestacional e crianças menores de dois anos com cardiopatia hemodinamicamente instável ou doença pulmonar crônica que receberam palivizumabe para profilaxia contra infecções graves pelo vírus sincicial respiratório, em 2008. No período do estudo, em cada episódio de infecção aguda do trato respiratório, coletou-se aspirado de nasofaringe para identificar vírus sincicial respiratório, adenovírus, parainfluenza 1, 2 e 3, influenza A e B por imunofluorescência direta, rinovírus e metapneumovírus por reação em cadeia de polimerase precedida de transcriptase reversa. Monitoraram-se internações e óbitos nesse grupo.

**RESULTADOS:**

Das 198 crianças acompanhadas, 117 (59,1%) apresentaram infecções agudas de trato respiratório, totalizando 175 episódios. De 76 aspirados de nasofaringe coletados na vigência de infecções do trato respiratório, 37 foram positivos, encontrando-se: rinovírus (75,7%), vírus sincicial respiratório (18,9%), parainfluenza (8,1%), adenovírus (2,7%), metapneumovírus (2,7%) e múltiplos agentes em três amostras. Das 198 crianças, 48 (24,4%) foram internadas, sendo 30 (15,2%) por etiologia não respiratória e 18 (9,1%) por problemas respiratórios; entre os 18 casos, um foi por vírus sincicial respiratório. Duas crianças evoluíram para óbito, não tendo sido identificado o vírus sincicial respiratório.

**CONCLUSÕES:**

Na vigência de profilaxia, observou-se frequência baixa de infecções pelo vírus sincicial respiratório e baixo índice de hospitalizações, sugerindo benefício da profilaxia com palivizumabe.

## Introdução

O vírus sincicial respiratório (VSR) é o principal agente de infecções agudas do trato respiratório inferior em crianças com menos de dois anos. Tem distribuição global e comportamento sazonal. Estima-se que, no mundo, 33,8 milhões de novos episódios de infecções agudas do trato respiratório inferior por VSR ocorram anualmente em menores de cinco anos. Destes, cerca de 3,4 milhões necessitam de internação hospitalar e 66 mil a 199 mil crianças evoluem para óbito, das quais 99% em países em desenvolvimento^(^ ^1^ ^)^.

Em geral, a infecção primária pelo VSR evolui como resfriado comum. Entretanto, aproximadamente 25% das crianças menores de dois anos podem apresentar, em seu primeiro episódio, infecção de vias aéreas inferiores, insuficiência respiratória grave, necessidade de assistência ventilatória e, ocasionalmente, óbito^(^ ^2^ ^)^.

Crianças prematuras com doença pulmonar crônica, imunodeprimidas e portadoras de cardiopatias (principalmente as cianóticas) apresentam maior risco de doença respiratória grave por VSR^(^ ^3^ ^-^ ^6^ ^)^. Alguns fatores ambientais também se associam à maior ocorrência de infecções pelo VSR, tais como exposição ao fumo, frequência às creches ou contato domiciliar com outras crianças^(^ ^4^ ^,^ ^5^ ^)^.

Em países tropicais e subtropicais, a ocorrência de infecções por VSR é maior no outono e no inverno, com variações regionais significantes^ (7)^. Estudos epidemiológicos do VSR na região Sudeste do Brasil mostraram que a infecção por VSR inicia-se em abril, com pico em maio e em junho^(^ ^2^ ^,^ ^8^ ^)^. Estudos mais recentes realizados na cidade de São Paulo mostraram que a estação de VSR ocorre de março a julho, podendo iniciar mais precocemente ou estender-se até agosto^(^ ^9^ ^,^ ^10^ ^)^. 

Não há tratamento específico para infecções por VSR^(^ ^7^ ^,^ ^11^ ^)^, sendo amplamente recomendada a profilaxia com anticorpo monoclonal específico contra o VSR (palivizumabe) para casos de alto risco^(^ ^4^ ^-^ ^6^ ^)^. Seu uso mostrou redução nas taxas de internação hospitalar por infecção por VSR (55%), de internações em UTI (57%), do tempo de internação (42%) e do número de dias com necessidade de oxigênio (40%)^(^ ^4^ ^,^ ^5^ ^,^ ^12^ ^,^ ^13^ ^)^. 

O palivizumabe é, no entanto, uma medicação de custo elevado. No Brasil, até 2007, muitas crianças receberam essa medicação após recursos judiciais, nem sempre com base em critérios técnicos^(^ ^14^ ^)^. A resolução normativa SS nº 249, de 13 de julho de 2007, da Secretaria de Saúde do Estado de São Paulo^(^ ^15^ ^)^, disponibilizou gratuitamente, no Sistema Único de Saúde, o palivizumabe para pacientes classificados como "altamente recomendados" pela Sociedade Brasileira de Pediatria^(^ ^5^ ^)^. Alguns centros foram indicados pela Secretaria do Estado como local para aplicação do palivizumabe e o Centro de Referência para Imunobiológicos Especiais (Crie) da Universidade Federal de São Paulo (Unifesp) foi o primeiro designado para o município de São Paulo. 

Nesse contexto, os objetivos do estudo foram descrever a etiologia dos quadros de infecção respiratória aguda viral em crianças submetidas à profilaxia com palivizumabe e analisar as taxas de internações e óbitos nesse grupo de crianças.

## Método

Estudo prospectivo com lactentes que receberam palivizumabe no Crie-Unifesp em 2008. O projeto foi aprovado pelo Comitê de Ética em Pesquisa da instituição, tendo sido solicitada a assinatura do termo de consentimento livre e esclarecido pelos pais/responsáveis.

Incluíram-se no estudo os lactentes com indicação de palivizumabe segundo a resolução normativa SS nº 249, de 13 de julho de 2007^(^ ^15^ ^)^, ou seja, crianças menores de um ano, nascidas com idade gestacional inferior a 29 semanas, após alta hospitalar, e crianças menores de dois anos portadoras de cardiopatia congênita com repercussão hemodinâmica ou com doença pulmonar crônica, que necessitaram de tratamento nos seis meses anteriores ao período de sazonalidade do VSR. 

A profilaxia iniciou-se em abril de 2008, incluindo-se os lactentes que receberam a primeira dose de palivizumabe até o último dia do mês de junho, na dose de 15mg/kg, via intramuscular (IM), no músculo vasto lateral da coxa, com intervalo de 30 dias e máximo de cinco doses. O agendamento foi realizado agrupando-se os pacientes duas vezes por semana para compartilhar o frasco do imunobiológico. 

Os dados sobre a história clínica da mãe e da criança e o motivo da indicação da profilaxia contra VSR foram coletados no dia da primeira dose do palivizumabe por meio de um questionário aplicado aos pais, além da análise do formulário médico que indicava a administração do anticorpo monoclonal.

Os pacientes foram acompanhados com consultas mensais durante todo o período de sazonalidade (abril a setembro) e os pais, orientados a comunicar a pesquisadora caso a criança apresentasse infecção aguda do trato respiratório nesse período (vigilância passiva). Além disso, uma vez por semana, cada paciente foi contatado por telefone para se buscarem intercorrências respiratórias e hospitalizações (vigilância ativa) durante todo período do estudo.

A infecção aguda do trato respiratório foi definida de acordo com a Organização Mundial da Saúde^(^ ^16^ ^)^ como presença ou não de febre, associada à coriza ou congestão nasal, dor de garganta, tosse, sibilância, taquipneia ou dificuldade respiratória.

Na vigência de quadro respiratório agudo, coletou-se um aspirado de nasofaringe (NPA) até o quarto dia após o início dos sintomas para pesquisa dos seguintes vírus respiratórios: influenza A e B, parainfluenza 1, 2 e 3, adenovírus, VSR, rinovírus e metapneumovírus. Para tais pacientes, aplicou-se um segundo questionário com dados clínicos sobre o evento respiratório atual. 

A taxa de internação pelo VSR foi calculada pela taxa de internação por doença respiratória, multiplicado pelo número de crianças internadas com teste positivo para VSR, dividido pelo número de crianças internadas por infecção respiratória e que realizaram a coleta^(^ ^13^ ^)^.

O aspirado de nasofaringe foi coletado com sonda nº 6 ou 8, introduzida na nasofaringe, após lavagem com soro fisiológico 0,9%. O aspirado foi colocado em frasco estéril com 5mL de soro fisiológico 0,9%. A amostra foi conservada sob refrigeração a 2-8°C e levada ao Laboratório de Virologia da instituição em, no máximo, duas horas. 

As amostras foram fracionadas, sendo uma alíquota centrifugada para realizar a imunofluorescência direta e a outra, congelada a -80°C, para teste molecular. Realizou-se a imunofluorescência direta para pesquisa de vírus influenza A e B, parainfluenza 1, 2 e 3, adenovírus e VSR, conforme o *Light Diagnostics**^(r)^* *Respiratory Screen Kit - Respiratory Panel 1 DFA* (Chemicon International, Inc.). Todas as amostras foram submetidas à reação em cadeia de polimerase precedida por transcriptase reversa para rinovírus e metapneumovírus humano^(^ ^17^ ^-^ ^19^ ^)^.

As variáveis numéricas com distribuição normal foram comparadas pelo teste *t* de Student e aquelas com distribuição não normal, pelo teste de Mann-Whitney. As variáveis categóricas foram comparadas pelo teste do qui-quadrado ou teste exato de Fisher. As análises estatísticas foram realizadas com o programa *Statistical Package for the Social Sciences* (SPSS), versão 17, sendo significante *p*<0,05. 

## Resultados

Em 2008, 210 lactentes foram submetidos à profilaxia com palivizumabe no Crie-Unifesp. Destes, nove foram excluídos por não preencherem os critérios de inclusão, um por não ter contato telefônico e dois porque foram transferidos para outro serviço antes do final do período de profilaxia. Desta forma, incluíram-se no estudo 198 lactentes.

As crianças avaliadas apresentaram média de idade gestacional de 30,2±5,1 semanas (variação de 23,0-41,2), peso ao nascer de 1482±900g (variação de 450-4165), mediana do tempo de ventilação mecânica de 12 dias (variação de 0-163; Q1 [1º quartil]-Q3 [3º quartil]: 2-40 dias) e ficaram internadas na unidade neonatal por 76±60 dias (variação de 2-638; Q1-Q3: 48-100 dias). A média de idade dos lactentes no momento da inclusão no estudo foi de 8,4±4,9 meses, sendo 52% do sexo masculino. 

Das crianças incluídas, 142 (71,7%) nasceram em hospital privado e 56 (28,3%), em hospital público. A mediana da renda familiar foi de R$ 2.500,00 (variação de R$ 300,00-20.000,00; Q1-Q3: R$ 1.050,00-5.000,00) com a seguinte classificação social, segundo a Associação Brasileira de Estudos Populacionais (ABEP - critério Brasil 2008): classe A1 - 5 (2,5%) famílias; A2 - 34 (17,2 %); B1 - 38 (19,2%); B2 - 57 (28,8%); C1 - 27 (13,6%); C2 - 29 (14,7%) e D - 8 (4,0%) famílias. 

A mediana do número de adultos no domicílio foi igual a 2 (variação de 0-7), de adolescentes foi de 0 (variação de 0-2) e de crianças, de 1 (variação de 1-4). O número de domicílios com fumantes foi de 57 (28,8%), sendo 22 (11,1%) mães fumantes. 

Com base nos critérios de indicação do palivizumabe, 29 (14,6%) crianças apresentavam idade gestacional <29 semanas sem displasia broncopulmonar; 53 (26,8%) eram portadoras de cardiopatia congênita hemodinamicamente instável, 116 (58,6%) apresentavam displasia broncopulmonar, sendo que 91 (78,4%) prematuros com displasia apresentavam idade gestacional <29 semanas.

Cada criança recebeu em mediana quatro doses de palivizumabe (variação de 1-5; Q1-Q3: 3-4 doses), totalizando 754 doses, com uso de 749,1mL do imunobiológico. Das 198 crianças acompanhadas, 45 (22,7%) receberam cinco doses de palivizumabe, 75 (37,9%) quatro doses, 75 (37,9%) três doses, 1 (0,5%) recebeu duas doses e 2 (1,0%) receberam uma dose. O intervalo entre as doses foi inferior a 35 dias em 96,9% dos casos da primeira para segunda dose, em 93,3% dos casos da segunda para a terceira dose, em 78,3% dos casos da terceira para quarta dose e em 60,0% dos casos da quarta para quinta dose. 

Em relação às intercorrências clínicas, 117 (59,1%) crianças tiveram pelo menos um episódio de infecção respiratória, perfazendo 175 episódios com mediana de 1,0 (variação de 0-4; Q1-Q3: 1-2 episódios/pacientes) e média de 1,5 episódios/paciente. Das infecções respiratórias, 104 (59,4%) foram infecção do trato respiratório superior e 71 (40,6%), do trato respiratório inferior (Figura 1). 

**Figura 1:** Incidência de infecção de trato respiratório superior e infecção de trato respiratório inferior em lactentes que receberam palivizumabe profilático, entre abril e setembro de 2008

Entre as crianças avaliadas, 48 (24,2%) necessitaram de internação, sendo 30 (15,2%) devido à etiologia não respiratória e 18 (9,1%) por problemas respiratórios. Das 18 internações por causa respiratória, 9 (50%) foram por pneumonia e 9 (50,0%) por bronquiolite, sendo que, destes, três evoluíram com pneumonia. A mediana do tempo de internação por essas doenças foi de 9 dias (variação de 3-28; Q1-Q3: 4-12 dias). 

Foram coletados NPAs em 13 (72,2%) dos 18 casos internados, dos quais 11 foram negativos (seis casos de bronquiolites e cinco pneumonias), em um caso foi detectado VSR (bronquiolite) e, em outro, identificou-se parainfluenza 2 e rinovírus (bronquiolite). A taxa de internação por VSR foi de 0,7% (18/198 *versus* 1/13), conforme cálculo descrito anteriormente. 

A comparação entre crianças com e sem bronquiolite não mostrou diferença estatística quanto ao sexo, peso ao nascer, idade gestacional, tempo de internação no período neonatal, dias de ventilação mecânica, frequência a creches e presença de fumantes no domicílio. Entretanto, crianças com bronquiolite tiveram menor taxa de aleitamento materno (*p*=0,021) e mais internações hospitalares (*p*<0,001) (Tabela 1). 

**Tabela 1:** Características demográficas e clínicas das crianças avaliadas, segundo a presença de diagnóstico de bronquiolite

No período de acompanhamento, ocorreram dois (1%) óbitos, um no pós-operatório de cirurgia cardíaca (hipoplasia de ventrículo esquerdo) e outro após internação por pneumonia em uma criança portadora de cardiopatia congênita, que evoluiu com choque séptico. Nesse paciente, a secreção de nasofaringe foi negativa para os vírus pesquisados.

Dos 175 episódios de infecção respiratória, coletaram-se 76 (43,4%) NPAs, sendo 37 (48,7%) negativos; em dois pacientes, o material coletado foi insuficiente e, em 37 (48,7%), detectou-se pelo menos um vírus. Os vírus identificados estão apresentados na Figura 2. Dos sete casos em que foram detectados VSR, dois ocorreram em abril, três em maio e dois em julho. 

**Figura 2:** Distribuição em porcentagem dos vírus encontrados no aspirado de nasofaringe em lactentes internados e não internados com quadro de infecção de vias aéreas

## Discussão

Este é o primeiro estudo brasileiro que avaliou infecções respiratórias e vírus envolvidos em crianças que receberam profilaxia com palivizumabe para VSR. 

Das 198 crianças seguidas no período de estudo, cerca de 60% tiveram pelo menos um episódio de infecção respiratória, com predominância de infecções agudas do trato respiratório superior, sendo resfriado comum o mais frequente. A infecção aguda do trato respiratório inferior mais frequente foi a bronquiolite. Das amostras de NPA coletadas na vigência de infecção respiratória, em metade dos casos detectou-se algum tipo de vírus, sendo o rinovírus o mais frequente, seguido pelo VSR. Nos casos em que foram identificados rinovírus, cerca de 1/3 apresentaram infecções agudas do trato respiratório inferior I com crise de subilância, confirmando-se os dados da literatura^(^ ^20^ ^)^ que mostram o rinovírus como importante causa de sibilância em menores de dois anos. Das sete crianças com VSR, seis (85,7%) tiveram quadro de bronquiolite e uma, de resfriado comum. Nenhum caso de influenza foi detectado. As crianças acima de seis meses e seus pais receberam a vacina influenza na estação avaliada.

Entre as crianças internadas por infecção respiratória, observou-se baixa taxa de internação por rinovírus, com apenas um caso de bronquiolite com coinfecção por rinovirus e parainfluenza 2, ao contrário do referido por Piotrowska *et al*, que encontraram taxa de internação de 55% entre os casos em que foram detectados rinovírus^(^ ^21^ ^)^. 

Os estudos sobre a incidência de VSR incluem crianças com grande variabilidade de faixa etária, região, população internada ou comunidade. Tal fato, associado à inexistência de estudos brasileiros com coortes de crianças de alto risco em uso de palivizumabe, dificulta a comparação com os dados encontrados nesta investigação. 

Neste estudo, a taxa de internação por doença respiratória em crianças que receberam palivizumabe foi de 9,1% e a taxa de internação por VSR foi de 0,7%. Tais dados mostram baixa taxa de internação por VSR na coorte que recebeu palivizumabe, em comparação com estudos com crianças de alto risco para infecção grave pelo VSR sem profilaxia. Estudo clássico multicêntrico com prematuros de alto risco observou taxa de internação por VSR de 10,6% no grupo sem profilaxia, com redução de 55% desses índices no grupo com profilaxia^(^ ^12^ ^)^. Em outro estudo com crianças menores de dois anos portadoras de cardiopatia congênita com repercussão hemodinâmica e que não receberam profilaxia, a taxa de internação por VSR foi de 9,7%^(^ ^3^ ^)^. Ao analisar estudos de crianças de alto risco que receberam palivizumabe, nossos achados são comparáveis. Boivin *et al**^(^* ^22^ ^)^ encontraram taxa de internação de 12,9% por infecções agudas de trato respiratório e de 0,9% por VSR em um grupo de crianças submetidas à profilaxia com palivizumabe. Paes *et al**^(^* ^23^ ^)^, avaliando crianças em uso de palivizumabe de 2005 a 2012 no Canadá, observaram taxa de internação por infecções agudas de trato respiratório de 6,6% e, por VSR, de 1,5%. 

Neste estudo, os casos de infecção por VSR ocorreram entre abril e julho. Dados epidemiológicos mostram que, em São Paulo, quando foi iniciada a profilaxia em abril, o VSR já estava em circulação, o que sugere que a profilaxia deva ser iniciada mais precocemente. Igualmente, estudos realizados com crianças internadas na cidade de São Paulo mostraram aumento na frequência de infecções por VSR de março a julho^(^ ^9^ ^,^ ^10^ ^)^. Das 198 crianças acompanhadas, 98,5% receberam três ou mais doses de palivizumabe, com intervalo adequado entre as doses até a terceira dose. Entretanto, houve atraso, em média, de 6,6 dias (variação de 1-15) no intervalo entre a quarta e a quinta dose, decorrente de atraso no fornecimento do palivizumabe pela Secretaria de Saúde de São Paulo no mês de agosto. As crianças que tiveram alta da unidade neonatal após o início da estação receberam menor número de doses, bem como aquelas que foram internadas durante o período da profilaxia, visto que o palivizumabe estava disponível apenas para crianças não internadas. Assim, se a profilaxia com palivizumabe fosse iniciada na estação apropriada também para prematuros internados na unidade neonatal, conforme recomendação da Sociedade Brasileira de Pediatria^(^ ^24^ ^)^, as crianças poderiam ter recebido maior número de doses. 

A estratégia de otimização da administração do palivizumabe com agendamento agrupado levou a uma economia de 28,2%, com uso de 749,1mL do palivizumabe, em comparação a 1.044mL que teriam sido utilizados em caso de agendamento individual^(^ ^25^ ^)^. 

Apesar do grande número de crianças, observou-se que a maior parte era proveniente de serviços privados e conveniados, provavelmente pelo maior acesso à informação dessas famílias, mostrando-se a necessidade de melhorar a divulgação do programa em todos os serviços de saúde. Este foi o primeiro ano do programa de profilaxia ao VSR no Estado de São Paulo e, segundo dados da Divisão de Imunizações da Secretaria de Saúde do Estado de São Paulo, em 2010, o número absoluto de pacientes atendidos no programa praticamente duplicou, sendo a procedência equitativa entre serviços públicos e privados^(^ ^26^ ^)^.

Em maio de 2013, uma nova portaria do Ministério da Saúde aprovou o protocolo de uso do palivizumabe para todo o território nacional, respeitando-se as diferenças sazonais de cada região. Foram contemplados prematuros com menos de um ano de idade, nascidos com idade gestacional menor ou igual a 28 semanas, além de crianças até dois anos de idade portadoras de doença pulmonar crônica ou cardiopatia congênita com repercussão hemodinâmica comprovada. Além disso, incluíram-se crianças internadas, administrando-se o palivizumabe, nesses casos, em ambiente hospitalar^(^ ^27^ ^)^.

 A falta de um grupo controle foi uma limitação deste estudo; entretanto, não seria ético incluir um grupo de crianças de mesma faixa etária, com características semelhantes às do grupo estudado, e não disponibilizar o anticorpo monoclonal. Também não foi possível coletar NPA de todos os casos de infecções respiratórias, principalmente nos pacientes com infecções agudas de trato respiratório superior. Houve uma limitação laboratorial em se realizar os exames nos finais de semana, o que dificultou a coleta, principalmente dos casos de infecções respiratórias de curta duração ou por motivo de viagens da família. 

Deste modo, em estação de alta circulação de VSR, o programa de profilaxia com palivizumabe em crianças de alto risco mostrou baixa frequência de infecções pelo VSR com baixa taxa de hospitalizações e nenhum óbito por esse agente, o que sugere o benefício da profilaxia com palivizumabe. Nas crianças em uso de palivizumabe, o principal vírus isolado nas infecções agudas de trato respiratório foi o rinovírus, com alto índice de sibilância e baixa taxa de internação, seguido pelo VSR.
